# Perception and Acceptance of COVID-19 Vaccine Among Healthcare Workers in Jeddah, Saudi Arabia

**DOI:** 10.7759/cureus.35673

**Published:** 2023-03-01

**Authors:** Ebtihal K Alfosail, Majed Alghamdi

**Affiliations:** 1 Preventive Medicine Program, Public Health Administration, Ministry of Health, Jeddah, SAU; 2 Preventive Medicine, Joint Program of Preventive Medicine, Jeddah, SAU

**Keywords:** health belief model, perception, health care workers, vaccination, covid 19

## Abstract

Introduction

The COVID-19 pandemic has resulted in significant hospitalizations and deaths, particularly among healthcare workers (HCWs). Various therapeutic and preventive measures have been introduced, with vaccination considered the primary preventive measure. This study aims to assess the perceptions and acceptance of COVID-19 vaccination among HCWs.

Methods

We conducted an analytical cross-sectional study on HCWs in hospitals located in Jeddah, Saudi Arabia. The study included physicians, nurses, pharmacists, lab technicians, and radiologists who work in the Ministry of Health's general hospitals. A total of 394 participants were included in the study. Data were analyzed using SPSS v26, and a p-value less than 0.05 was considered significant.

Results

A majority of the participants (72.6%) were female, aged between 31-40 years (55.3%), and married (59.6%). More than half of the participants (55.6%) had received training on dealing with COVID-19. The mean scores for COVID-19 vaccine refusal, perceived susceptibility, perceived severity, perceived benefits, perceived barriers, and perceived effectiveness of COVID-19 vaccination were 18.36 ± 7.02, 14.48 ± 3.62, 11.51 ± 2.99, 12.39 ± 3.5, 8.25 ± 2.35, and 8.40 ± 2.46, respectively. Age was found to be correlated with the perceived severity of COVID-19 in non-vaccination (p=0.048), while gender was associated with the perceived severity of COVID-19 (p=0.015). Marital status (p=0.001), years of experience (p=0.009), profession (p=0.019), and education (p=0.028) were found to be correlated with perceived susceptibility. Education levels were found to be correlated with the perceived benefits of vaccination (p=0.007), perceived barriers to vaccines (p=0.002), and vaccine views (p=0.002). Years of experience (p=0.017) were found to be correlated with perceived severity of COVID-19, while profession type was significantly associated with perceived severity of COVID-19 (p=0.016) and vaccine view (p=0.008)

Conclusion

The study found that participants had a positive perception and high acceptance of COVID-19 vaccination. The results also indicated that various sociodemographic factors were associated with the perception and acceptance of COVID-19 vaccines among HCWs. These findings could help in formulating effective strategies to improve vaccination uptake rates among HCWs, thereby reducing transmission and mortality among Health Care Workers due to COVID-19.

## Introduction

The World Health Organization (WHO) has designated the outbreak of coronavirus disease of 2019 (COVID-19) as a public health emergency of international concern since January 30, 2020 and as a pandemic since March 11, 2020. This disease is caused by the severe acute respiratory syndrome coronavirus-2 (SARS-CoV-2) [1], and it has had a significant impact on global health and the economy. As of January 17, 2023, there have been 662,735,182 confirmed cases of COVID-19, resulting in 6,706,305 deaths worldwide [2]. Despite several therapeutic options being used to manage COVID-19, the number of cases and fatalities continued to rise while vaccines were being developed. Companies conducted clinical trials to provide a safe and effective vaccine. The Saudi Food and Drug Administration (FDA) has approved the use of Pfizer (BioNTech), Moderna, and AstraZeneca vaccines. However, high levels of vaccine hesitancy have been reported in Saudi Arabia and other countries due to misconceptions surrounding COVID-19 and its vaccines [3-5]. As anti-vaccination groups became more active, particularly after the quarantine application, conspiracy theory myths became prevalent during the early days of the pandemic.

Healthcare workers (HCWs) play a critical role in influencing acceptance, agreement, perception, awareness, and knowledge of COVID-19 vaccines among their patients and the general public [6]. Despite their influence on society, HCWs are under a lot of pressure for being directly exposed as the first-line responders to the pandemic, which can affect their attitudes and beliefs toward vaccinations and their motivation for accepting COVID-19 vaccines. COVID-19 vaccination campaigns began on 17 December 2020 in Saudi Arabia, together with awareness campaigns to persuade and educate the population about the importance of COVID-19 vaccination [7]. COVID-19 vaccination has been made available in Saudi Arabia in three priority categories, with the first stage involving elderly individuals and all healthcare providers. Saudi Arabia mandated COVID-19 vaccines, allowing only fully vaccinated citizens and residents to enter all places, activities, events, government and private facilities, or use public transportation across the Kingdom's regions [8]. Globally, acceptance of COVID-19 vaccines among HCWs varies widely, ranging from 27.7% in the Republic of the Congo to 78.1% in Israel [9]. In some Asian countries, namely China, Indonesia, and Malaysia, a higher level of acceptance among the general population (90%) was reported [10-12]. However, studies have shown that the acceptance rate decreased over time, decreasing from 85.8% in April 2020 to 75.8% after one year among parents in Australia [13,14]. Similar declines in acceptance rates were reported in Europe [15].

Barry et al. [16] found a 70% acceptance rate among HCWs in Saudi Arabia. In contrast, Alfageeh et al. [17] found a low acceptance rate of 48% among 2319 participants of the general population through a web-based survey, consistent with another study conducted by Magadmi and Kamel [18]. Therefore, this study evaluated the perception and acceptance of COVID-19 vaccination among HCWs in Jeddah, Saudi Arabia.

## Materials and methods

An analytical cross-sectional study was conducted between June and December 2022 on HCWs in Jeddah city. The study included physicians, nurses, pharmacists, lab technicians, and radiologists who work in the Ministry of General Health hospitals. To achieve a confidence level of 95% and a statistically significant p-value of <0.05, a minimal sample size of 394 was required from a total of 18,343 HCWs in Jeddah. This was calculated using Raosoft (http://www.raosoft.com/samplesize.html).

A multistage sampling technique was employed to select the participants. The hospitals in Jeddah were stratified into two strata, with three hospitals in one stratum and two hospitals in another. One hospital was randomly selected from each stratum, and HCWs were proportionally selected from each hospital using convenience sampling. A validated questionnaire, which employed the health belief model, was adopted from a previous study [19]. A typical 5-point Likert scale was used as the numerical value assigned to each response option in a Likert scale. For example, a score of 1 for “strongly disagree” and a score of 5 for “strongly agree.” These scores are used to quantify participants' attitudes or opinions and allow for statistical analysis of the data. The hospital unit managers assisted the investigators in distributing the questionnaires to HCWs. Confidentiality was ensured by the anonymity of the questionnaires, and the data collected were solely used for the purposes of this study. HCWs were informed about the aim and objectives of the study and invited to participate after providing written consent. Ethical approval was obtained from the research committee at the Joint Program of Preventive Medicine in Jeddah, Saudi Arabia (Ethical approval code: A01416).

The data were analyzed using Statistical Package of Social Science (SPSS) version 26. Descriptive statistics, such as frequencies and percentages, were used to present nominal and ordinal data. Mean, median, and standard deviation or range were used to describe numerical variables. The means of domain scores were compared using T-test and ANOVA. A p-value < 0.05 was considered statistically significant.

## Results

This study included 394 participants aged 18-64 years. The majority of participants (218, 55.3%) were in the age group of 31-40 years. Most participants were female (286, 72.6%) while only 108 (27.4%) were male. In terms of marital status, 235 (59.6%) were married, 139 (35.3%) were single, 18 (4.65%) were divorced, and 2 (0.5%) were widows. The majority of participants were Saudi nationals (95.2%), and most had a bachelor's degree (232, 58.9%). Table 1 provides a detailed breakdown of the participants' demographics.

**Table 1:** Demographic characteristics of the participants (n=394)

Characteristics	Frequency	Percentages
Age group		
18-30 years	120	30.5
31-40 years	218	55.3
41-50 years	35	8.9
51-60 years	15	3.8
>60 years	6	1.5
Gender		
Male	108	27.4
Female	286	72.6
Marital status		
Divorced	18	4.6
Married	235	59.6
Single	139	35.3
Widow	2	0.5
Nationality		
Saudi	375	95.2
Non- Saudi	19	4.9
Education status		
Bachelor	232	58.9
Diploma	73	18.5
Graduate studies	89	22.6
Residence		
Madina	383	97.2
Outside Madina	11	2.8
Years of experience		
<1 years	86	21.8
1-4 years	40	10.2
5-9 years	115	29.2
>10 years	153	38.8

Table 2 presents detailed information on participants' work-related characteristics. More than half of the participants (55.6%) had received training on dealing with COVID-19. The majority of participants (61.4%) were seeing less than 50 patients per week, while only 8.9% were dealing with more than 200 patients per week. Most participants (73.9%) had an eight-hour shift, and the majority (68.8%) had morning shifts. Additionally, almost 90% of the participants were working in tertiary care hospitals.

**Table 2:** Health facility and work detail of the participants (n=394)

Variables	Frequency	Percentage
Training in dealing Covid 19	219	55.6
Patient per week		
<50	242	61.4
51-100	86	21.8
100-200	31	7.9
>200	35	8.9
Working hours per day		
8 hours	291	73.9
8- 12 hours	89	22.6
>12 hours	14	3.6
Work shift		
Morning	271	68.8
Evening	24	6.1
Mixed	99	25.1
Type of health facility		
Tertiary hospital	353	89.6
PHC	16	4.1
Other	25	6.3

In terms of participants' response regarding COVID infection, the majority were neutral, 111 (28.2%), when asked if they were afraid of infection. Similarly, 119 (30.2%) had a neutral opinion regarding feeling uncomfortable when thinking about infection. 190 (48.2%) strongly disagreed with the opinion that when thinking about the Coronavirus disease, they feel a sudden cold and wetness on their hands. More than 66% disagreed with the statement “I am afraid of losing my life due to the Coronavirus disease,” 56% disagreed with getting worried on watching COVID-19 news, and more than 80% disagreed with sleep disturbance due to fear of COVID-19 (Table 3).

**Table 3:** Response of participants with COVID-19 infection (n=394)

Response to infection	Strongly Agree	Agree	Neutral	Disagree	Strongly Disagree
I'm afraid of the Coronavirus disease	24(6.1)	99(25.1)	111(28.2)	101(25.6)	59(15.0)
I feel uncomfortable when I think about the Coronavirus disease	18(4.6)	88(22.3)	119(30.2)	101(25.6)	68(17.3)
When I think about the Coronavirus disease, I feel a sudden cold and wetness on my hand.	4(1.0)	19(4.8)	60(15.2)	121(30.7)	190(48.2)
I am afraid of losing my life due to the Coronavirus disease	8(2.0)	33(8.4)	89(22.6)	131(33.2)	133(33.8)
I feel more anxious and worried when watching the news and stories about the Coronavirus disease on social media	14(3.6)	50(12.7)	107(27.2)	118(29.9)	105(26.6)
I can't even sleep comfortably because I might get the Coronavirus disease	12(3.0)	16(4.1)	49(12.4)	122(31.0)	195(49.5)
When I think that I might get the Coronavirus disease, my heart rate (heart rhythm) rises sharply (I feel like it's pounding, fluttering, or beating irregularly)	10(2.5)	27(6.9)	62(15.7)	128(32.5)	167(42.4)

On the Likert scale, the overall mean score for the perceived severity of COVID-19 vaccination was 18.36 ± 7.02, with a minimum score of 6 and a maximum score of 30. Of all participants, 42.9% disagreed with the statement: “I feel that without this service, I won't be able to return to my workplace,” while 37% agreed with the statement. Moreover, 42% agreed with the statement, “I feel that without this service, my chances of getting a job will be affected.” 44% felt that without being vaccinated, they wouldn't be able to book face-to-face appointments with patients and coworkers, 43.9% felt that without being vaccinated, they wouldn't be able to go to the theater/movies/sports events, 61.9% felt that without being vaccinated they wouldn't be able to travel internationally, and 52% felt that without being vaccinated they would not enjoy the same liberties they did before the pandemic.

The mean score for perceived susceptibility to COVID-19 was 14.48 ± 3.62, with a minimum of 4 and a maximum of 20. 55.6% of the participants agreed that they were at risk of getting COVID-19 (SARS-CoV-2), 71.3% agreed that it is likely that they would get COVID-19, 61.7% agreed that individuals in their household were at risk for getting COVID-19 (SARS-CoV-2), and 61.2% felt knowledgeable about their risk of getting COVID-19 (SARS-CoV-2)

Regarding perceived COVID-19 severity, the mean score was 11.51 ± 2.99, with a minimum score of 4 and a maximum score of 20. Around a half (52%) of the participants believed that COVID-19 (SARS-CoV-2) was a severe health problem in general, 27.2% agreed that if they got COVID-19 (SARS-CoV-2), they would get sick, while 70.6% disagreed that if they got infected, they would die. Half (51.5%) disagreed that if they got COVID-19 (SARS-CoV-2) other members of their household would get sick.

Regarding the perceived benefits of COVID-19 vaccines, the mean score of benefit was 12.39 ± 3.5, with a minimum of 4 and a maximum of 20. Of all participants, 38.8% agreed that vaccination would make them feel safe only if immunity is obtained through a complete course of vaccination, 35.9% disagreed that the vaccine would make them feel safe only if immunity is obtained through a past COVID-19 (SARS-CoV-2) infection. Moreover, 47.2% agreed that the vaccine would facilitate economic recovery, and 55.9% agreed that the vaccine would facilitate social gatherings in closed spaces without restrictions (e.g., wearing masks, and limits on the number of people who can gather).

Regarding perceived barriers to vaccination, the mean score was 8.25 ± 2.35, with a minimum of 3 and a maximum of 15. The majority of participants, 60.7%, disagreed that their data on vaccination status would be passed on to third parties without their consent or commercialized, 58.9% disagreed that vaccination would be difficult for them to use if available only on smartphones/tablets, and 72.4% disagreed that vaccination would be difficult for them to access. Similarly, regarding vaccine views of participants on vaccination effectiveness, the mean score was 8.40 ± 2.46, with a minimum of 3 and a maximum of 15. Of all participants, 45% disagreed that they were not convinced that the vaccine would protect them against COVID-19 (SARS-CoV-2), 47.2% disagreed that they felt worried about people who had received a non-Saudi approved vaccine entering the country, and 48.3% agreed that they felt less at risk from catching coronavirus if they were around people who had been fully vaccinated. Details are shown in Table 4. 

**Table 4:** Participant perceptions and attitudes toward COVID-19 vaccination

Perceived severity of consequences of not taking the vaccine	Strongly Agree	Agree	Neutral	Disagree	Strongly Disagree
I feel that without this service, I won't be able to return to my workplace	43(10.9)	108(27.4)	74(18.8)	89(22.6)	80(20.3)
I feel that without this service, my chances of getting a job will be affected.	56(14.2)	111(28.2)	71(18.0)	70(17.8)	86(21.8)
I feel that without this service, I won't be able to book face-to-face appointments with my patients and coworkers	55(14.0)	121(30.7)	72(18.3)	65(16.5)	81(20.6)
I feel that without this service, I won't be able to go to the theatre/movies/sports events	56(14.2)	117(29.7)	62(15.7)	75(19.0)	84(21.3)
I feel that without this service, I won't be able to travel internationally	99(25.1)	145(36.8)	46(11.7)	42(10.7)	62(15.7)
I feel that without this service, I will not enjoy the same liberties I did before the pandemic	69(17.5)	136(34.5)	51(12.9)	54(13.7)	84(21.3)
Perceived COVID19 Susceptibility					
I am at risk of getting COVID-19 (SARS-CoV-2).	71(18.0)	148(37.6)	90(22.8)	53(13.5)	32(8.1)
It is likely that I will get COVID-19 (SARS-COV-2).	101(25.6)	180(45.7)	73(18.5)	23(5.8)	17(4.3)
Individuals in my household are at risk of getting COVID-19 (SARS-COV-2).	74(18.8)	169(42.9)	95(24.1)	29(7.4)	27(6.9)
I feel knowledgeable about my risk of getting COVID-19 (SARS-COV-2).	78(19.8)	163(41.4)	98(24.9)	37(9.4)	18(4.6)
Perceived COVID19 Severity					
I believe that COVID-19 (SARS-COV-2) is a severe health problem in general.	56(14.2)	149(37.8)	109(27.7)	58(14.7)	22(5.6)
If I get COVID-19 (SARS-COV-2), I will get sick.	18(4.6)	89(22.6)	151(38.3)	92(23.4)	44(11.2)
If I get COVID-19 (SARS-COV-2), I will die.	8(2.0)	18(4.6)	86(21.8)	94(23.9)	188(47.7)
If I get COVID-19 (SARS-COV-2), other members of my household will get sick.	37(9.4)	52(13.2)	102(25.9)	140(35.5)	63(16.0)
Perceived Benefits					
This service will make me feel safe only if immunity is obtained through the complete course of vaccination.	35(8.9)	114(28.9)	116(29.4)	76(19.3)	53(13.5)
This service will make me feel safe only if immunity is obtained through past COVID-19 (SARS-CoV-2) infection.	19(4.8)	67(17.0)	127(32.2)	105(26.6)	76(19.3)
This service will facilitate economic recovery.	70(17.8)	116(29.4)	120(30.5)	45(11.4)	43(10.9)
This service will facilitate social gatherings in closed spaces without restrictions (e.g., wearing masks, and limits on the number of people who can gather).	70(17.8)	150(38.1)	95(24.1)	48(12.2)	31(7.9)
Perceived Barriers to taking vaccination					
I'm afraid that my data will be passed on to third parties without my consent or commercialized.	10(2.5)	59(15.0)	86(21.8)	130(33.0)	109(27.7)
This service will be difficult for me to use if available only on smartphones/tablets.	15(3.8)	46(11.7)	101(25.6)	119(30.2)	113(28.7)
This service will be difficult for me to access.	10(2.5)	24(6.1)	75(19.0)	122(31.0)	163(41.4)
Vaccine Views					
I am not convinced that the vaccine will protect me against COVID-19 (SARS-CoV-2).	37(9.4)	82(20.8)	98(24.9)	107(27.2)	70(17.8)
I feel worried about people who have received a non-Saudi-approved vaccine entering the country.	21(5.3)	49(12.4)	138(35.0)	116(29.4)	70(17.8)
I feel less at risk from catching the coronavirus if I’m around people who have been fully vaccinated.	54(13.7)	97(24.6)	119(30.2)	65(16.5)	59(15.0)

The results of the analysis showed that age was a significant factor in the perceived severity of covid infection in patients who are not vaccinated (p=0.048). Gender was significantly associated with the severity of COVID-19 (p=0.015). Marital status (p=0.001), years of experience (p=0.009), profession (p=0.019), and education (p=0.028) were significantly associated with perceived susceptibility. Education was associated with perceived benefits of vaccination (p=0.007), barriers to vaccines (p=0.002), and vaccine views (p=0.002). Years of experience (p=0.017) were significantly associated with the perceived severity of COVID-19, and profession was significantly associated with the perceived severity of COVID-19 (p=0.016) and vaccine view (p=0.008) (Table 5).

**Table 5:** Comparison of demographic characteristics with the mean value of study parameters *ANOVA, ^ t-test

	Perceived severity non-vaccination		Perceived susceptibility		perceived severity of COVID-19		Perceived Benefits		Perceived Barriers		Vaccine Views	
	Mean square	P-value	Mean square	P-value	Mean square	P-value	Mean square	P-value	Mean square	P-value	Mean square	P-value
Age group	117.46	0.048*	34.41	0.322	12.52	0.234	23.78	0.117	13.67	0.120	13.40	0.065
Gender	16.29	0.566	1.052	0.777	52.85	0.015^	48.22	0.053	0.099	0.909	0.788	0.720
Marital status	8.06	0.922	70.48	0.001*	22.44	0.057	6.21	0.697	0.400	0.984	1.80	0.82
Nationality	178.05	0.27	10.29	0.457	8.32	0.397	31.08	0.090	10.69	0.240	5.05	0.438
Education status	58.67	0.276	46.65	0.028*	5.56	0.540	63.40	0.007*	45.73	0.002*	36.29	0.002*
Years of experience	74.11	0.212	50.08	0.009*	30.43	0.017*	21.25	0.176	5.92	0.499	12.84	0.096
Profession	60.28	0.286	31.16	0.019*	21.83	0.016*	23.41	0.078	15.44	0.042	16.41	0.008*

## Discussion

This study investigated the perception and acceptance of Coronavirus Disease 2019 (COVID-19) vaccination and its determinants among HCWs in Jeddah, Saudi Arabia. The findings showed that the Ministry of Health was the main source of information about COVID-19 for around three-quarters. This contrasts with the findings of other studies showing that media and social media were the primary sources of information about COVID-19 for the general public [11,17,20,21], which is expected since HCWs have direct access to trusted information from professionals and trusted health institutions such as the Ministries of Health. The fact that research has indicated that information from health institutions is associated with more willingness to comply with preventive measures for COVID-19 than other sources [21,22] confirms the trustworthiness and highlights the need for health institutions to adopt other channels, such as social media, to reach a wider audience and counteract false information on social media. 

Even though most participants got information from the Ministry of Health, the readership of scientific articles, which are the source of evidence-based information, was very low, in line with other previous studies [20,23]. This could be due to the high workload of HCWs that prevents them from sparing time searching articles. The rate of using articles as the source is even lower than that reported in a study conducted on Jordanian university students (9% vs. 24%) [23], which might be explained by the eagerness of students to explore more for their success or the huge trust in the Ministry of Health by HCWs making them feel satisfied with the information given.

Generally, participants perceived COVID-19 vaccines as beneficial, consistent with other previous studies [24]. They had a good perception of COVID-19 vaccines, aligning with previous research [6,25]. This might be due to access to accurate information by HCWs, as supported by other studies that showed that being an HCW and working in the healthcare sector was associated with high knowledge, good attitude, and high acceptability of COVID-19 vaccines [6,25,26].

We found that gender and age were associated with the perceived severity of COVID-19, which is consistent with previous studies. Giordani et al. [27] reported that women perceived COVID-19 as more life-threatening than men, and this perception was more pronounced among women aged 30-59 and 60 or older. Similarly, another study found that women were more anxious about COVID-19 than men [28]. Our findings also showed that marital status, education, years of experience, and profession type were significantly associated with perceived susceptibility to COVID-19 and vaccine refusal, indicating that demographic factors may influence attitudes toward vaccination. These results are consistent with other studies that have reported associations between vaccination willingness and factors such as profession type, education level, and marital status [12]. Previous research has suggested that being married, highly educated, experienced, or a doctor is all associated with better perception, attitude, and willingness to take up COVID-19 vaccines [4,9,10,12,16,29,30], which is in line with our findings. It is possible that these demographic characteristics are linked to higher levels of knowledge, responsibility, and concern for family, which may contribute to greater acceptance of vaccines. Further research is needed to explore these relationships in more detail among HCWs in Saudi Arabia.

This study's limitations were that a cross-sectional design cannot identify the causal effect on outcomes. The data collection was done using questionnaires prone to over- and under-reporting. The small sample size may lead to the inability to generalize the findings. Therefore, in-depth prospective and longitudinal studies are recommended.

## Conclusions

This study showed that the HCWs generally had a good perception and acceptance of COVID-19 vaccination, which may be explained by access to factual information from trusted sources, mainly the ministry of health. Moreover, age, gender, marital status, education, work experience, and vaccine views significantly influenced COVID-19 acceptance and perception among participants. These findings imply that demographic factors impact how people view and perceive COVID-19 vaccinations, highlighting the need for further studies to inform strategies to fight against COVID-19.
